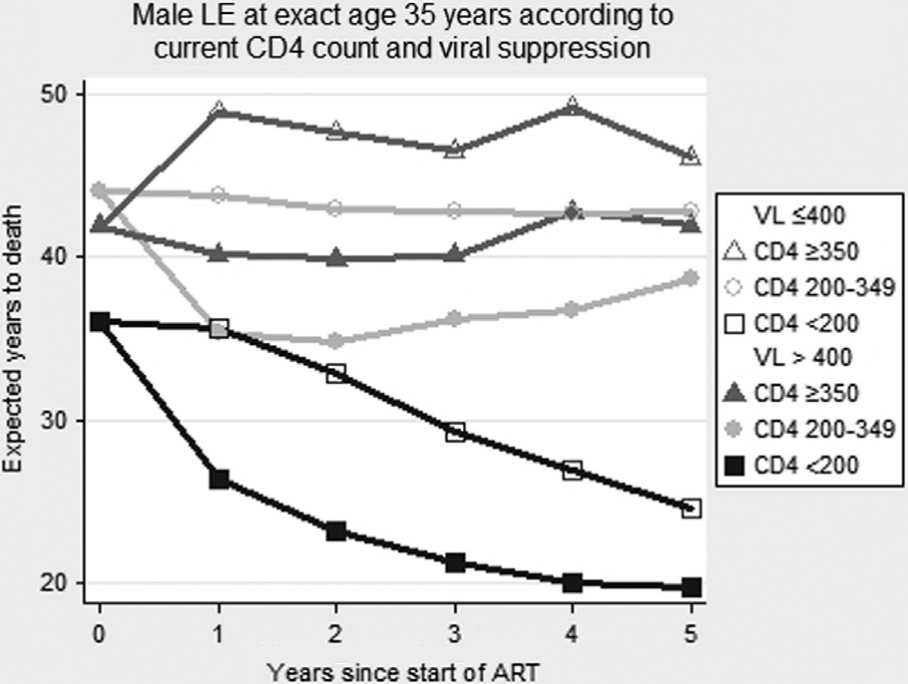# Life expectancy of HIV-1-positive individuals approaches normal conditional on response to antiretroviral therapy: UK Collaborative HIV Cohort Study

**DOI:** 10.7448/IAS.15.6.18078

**Published:** 2012-11-11

**Authors:** M May, M Gompels, C Sabin

**Affiliations:** 1 University of Bristol, Bristol, UK; 2 North Bristol NHS Trust, Bristol, UK; 3 University College London, Medical School, London, UK

## Abstract

Life expectancies (LEs) of patients in UK Collaborative HIV Cohort (UK CHIC) stratified by CD4 count at start of antiretroviral therapy (ART) have been estimated [1] but not gains in years of life in response to ART. We estimated LE associated with attained CD4 count and viral suppression at different durations of ART. Patients in UK CHIC aged > 20 years who started ART in 2000 to 2008 (excluding person who injects drugs) were followed to end of 2010. All-cause mortality was ascertained from clinic notes and by linkage to national records. We used the nearest CD4 count before ART and the last in each of years 1 to 5 of ART and determined whether patients were virally suppressed (HIV-1 RNA < 400 copies/mL) in the past year for those remaining under follow-up. Poisson models were used to estimate mortality rates by sex, age, latest CD4 count (<200, 200 to 349,≥350) and viral suppression for each duration of ART. Abridged life tables were constructed from age-specific mortality rates to estimate LE for ages 20 to 85 years. Results are presented as the average number of years that will be lived after exact age 35 years. A total of 17,021 patients started ART from 2000 to 2008 of whom 708 (4.2%) died; 3956 (23%) were lost to study follow-up. There was no difference in mortality between those with attained CD4 350 to 499 and ≥ 500. On starting ART, male LE at exact age 35 was 36, 44 and 42 (female LE 38, 46 and 44) years for attained CD4 < 200, 200 to 349,≥350, respectively; after 5 years on ART, it was 22, 42 and 46 (female LE 27, 46 and 51) years, respectively. Only 17% of patients had CD4 ≥ 350 at ART start, compared with 78% of patients on ART for > 5 years. The difference in LE between suppressed versus unsuppressed patients was around 11 years. The figure shows that both CD4 count and viral suppression contribute to changes in LE. Male patients that increased their CD4 in the 1st year of ART from < 200 to 200–349 or ≥ 350 gained 6 and 11 years of LE to 42 and 48 years, respectively, with similar rises for women. Overall, LE was 4 years greater for those on ART for > 5 years compared with those starting ART. Individuals that attain viral suppression and a CD4 count > 350 within 1 year of ART start have a normal LE with 35-year olds estimated to live to over 80 years on average. LE in patients with CD4 count < 200 beyond 5 years on ART drops by 15 years. Estimated LE may be biased by under-ascertainment of deaths, missing CD4 measurements and extrapolation beyond available data.

**Figure:**